# Can Systems Biology Understand Pathway Activation? Gene Expression Signatures as Surrogate Markers for Understanding the Complexity of Pathway Activation

**DOI:** 10.2174/138920208785133235

**Published:** 2008-08

**Authors:** Hiraku Itadani, Shinji Mizuarai, Hidehito Kotani

**Affiliations:** Department of Cancer Research, Banyu Tsukuba Research Institute, Merck Research Laboratory, Tsukuba, Ibaraki 300-2611, Japan

**Keywords:** Expression signature, signaling pathway, drug discovery, cancer therapy, systems biology.

## Abstract

Cancer is thought to be caused by a sequence of multiple genetic and epigenetic alterations which occur in one or more of the genes controlling cell cycle progression and signaling transduction. The complexity of carcinogenic mechanisms leads to heterogeneity in molecular phenotype, pathology, and prognosis of cancers.

Genome-wide mutational analysis of cancer genes in individual tumors is the most direct way to elucidate the complex process of disease progression, although such high-throughput sequencing technologies are not yet fully developed. As a surrogate marker for pathway activation analysis, expression profiling using microarrays has been successfully applied for the classification of tumor types, stages of tumor progression, or in some cases, prediction of clinical outcomes. However, the biological implication of those gene expression signatures is often unclear.

Systems biological approaches leverage the signature genes as a representation of changes in signaling pathways, instead of interpreting the relevance between each gene and phenotype. This approach, which can be achieved by comparing the gene set or the expression profile with those of reference experiments in which a defined pathway is modulated, will improve our understanding of cancer classification, clinical outcome, and carcinogenesis. In this review, we will discuss recent studies on the development of expression signatures to monitor signaling pathway activities and how these signatures can be used to improve the identification of responders to anticancer drugs.

## INTRODUCTION

In human cancer development, the accumulation of genetic mutations by DNA damage, cellular stress, and aging causes the deregulation of key molecules involved in cell signaling cascades, which leads to hyperactivate or inactivate signaling pathways. This deregulation results in accelerated and uncontrolled cell cycle progression triggering tumor initiation and growth [1].

Somatic mutations in human cancers have been systematically surveyed in recent years by a high-throughput genotyping technique [2-5]. According to a study in which 13,000 genes in human cancers were sequenced, each tumor accumulated 90 mutations on average, although the majority of them are likely to be unrelated to cancer development [6]. Understanding pathway deregulation in cancer cells and the identification of biomarkers to monitor oncogenic signaling activity are essential for the development of targeted cancer therapy.

Although detecting mutations in all cancer genes is a straightforward approach to measure oncogenic pathway activities, current genotyping techniques are still not suitable for this purpose. In addition, multiple and complex mutations of cancer-related genes across the several signaling pathways make it difficult for us to determine which pathway is activated and dominantly contributes to cancer progression. Considering that most cellular signaling pathways are regulated at the protein level, such as by phosphorylation/dephosphorylation, proteomics markers might more directly reflect the pathway status [7]. However, despite requiring simultaneous analyses of multiple proteins to accurately determine the activation status of an oncogenic pathway, current technology does not allow us to do multiplex assays, especially in clinical samples. Alternatively, the identification of a set of genes whose expression pattern represents the status of an oncogenic pathway can serve as a surrogate marker to monitor their activity. Owing to microarray technology, which has become more sophisticated in the last ten years with higher sensitivity, selectivity, and reproducibility compared to other Omics technologies, various signaling pathway signatures have been developed. Moreover, since the activation of most oncogenic signaling results in the transactivation of genes mediated by a key transcription factor, it would be reasonable to use an expression signature as a surrogate biomarker to monitor oncogenic pathway activation.

## GENE EXPRESSION SIGNATURE AS A PATHWAY MARKER

Recently, an increasing number of expression profiling studies in oncology have been performed in order to elucidate signaling pathway activation mechanisms in cancer cells (Table **1**). Activation or inactivation of signaling pathways affects gene expressions *via *downstream transcription factors and their regulatory genes, for example β-catenin/TCF in the Wnt pathway and oncogenic Myc in the RAS pathway. Thus, the pattern of gene expression changes observed in cancer cells is a reflection of intracellular signaling activities involved in carcinogenesis and/or drug sensitivity.

Although one of the intrinsic problems of microarray analysis is that a signature gene set contains some false positives by nature of high throughput, current advances in controlling the false discovery rate have overcome this problem to some extent, solidifying the status of expression profiling as the gold standard among non-biased genome wide approaches [8]. However, discovering the biological connection between genes identified by microarray and their phenotypic effect remains elusive. Several analytical tools have been developed to help us understand the biology of the signature, such as GoMiner [9] and the gene set enrichment analysis (GSEA) [10,  11], which are used to find which annotations are enriched in a set of genes. Pathway analysis tools, for example Ingenuity and MetaCore, are often used to identify relationships among the signature genes, such as activation, inhibition, and binding [12-14]. The problem with these types of analyses is that stand-alone analysis of molecular profiling does not give us information about which pathways are activated or inactivated under specific conditions.

**Fig. (1):**
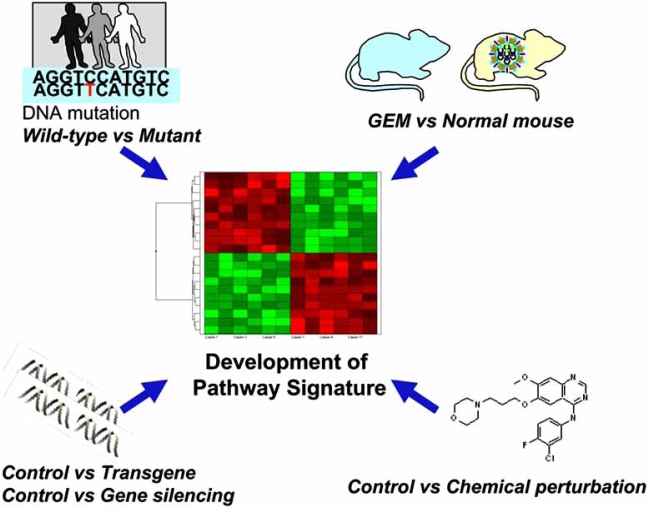
Methods to develop pathway signatures. Gene expression regulated by signaling pathways can be detected by comparing pathway-activated *vs*. quiescent cells or baseline-level *vs*. inactivated cells using gene expression microarrays (See text for detail).

Accumulating data for pathway signatures is expected to address the issue on how to measure pathway activity by expression profiling (Fig. **1**). In this review, we define a pathway signature as a set of genes whose expression changes are correlated with the activity of a signaling pathway. Ideally, a pathway signature should be regulated by only one specific pathway. If such genes exist for each pathway, we can detect signaling perturbation in any samples regardless of tissue type and species. Practically, the expression of any given gene is affected by more than one pathway. Therefore, the development of signatures, which are robust in as many tissue types as possible, is preferable. In order to estimate pathway status, the expression pattern of the pathway signature in the query profile is tested for whether the anti-correlation between up and down signatures is statistically significant or not. The advantage of using a pathway signature to predict pathway activation is that we will be able to identify functional defects in the signaling pathway, while genetic mutation does not necessarily lead to the functional deregulation. Another advantage of this approach is that we do not need to know the biological relevance of each signature gene to the pathway. Rather, statistically accurate predictions are more crucial to practically use the pathway signature.

## STRATEGY TO DEVELOP PATHWAY SIGNATURES

### Comparison of Expression Profiles between Mutant *vs*. Wild-Type Cells

Pathway signatures are commonly developed by comparing gene expression levels between baseline samples and activated samples. Examples include the comparison between tumors harboring a gene mutation vs. those without the mutation, and cell lines with and without exogenous gene expression. Many mutations are known to lead to deregulated activation of one or more oncogenic pathways, followed by the transactivation of downstream genes which will be identified as a pathway signature. For example, the somatic mutation V599E in BRAF, commonly observed in melanoma, is known to activate the MEK-ERK cascade constitutively [15,  16]. Expression profiling of melanoma cell lines with or without the BRAF mutation revealed signature genes which allowed for the classification of cells as mutant or wild-type [17]. 

The signature discovery method based on mutation status can be applied not only to mutations which activate a specific pathway, but also to those which inactivate the pathway. Mutations in the tumor suppressor gene p53 usually cause a defect in transactivating its regulatory genes in response to cellular stress or DNA damage [18,  19]. By comparing the expression profiles of p53 mutant and wild-type breast cancers, 32 signature genes differentially regulated between the two groups were identified. It was shown that the p53 classifier outperformed DNA sequence based p53 determination in predicting prognostic significance and drug response [20]. Instead of gene mutations, the detection of protein expression levels has also been used to measure pathway activity. Protein expression levels of key molecules in a pathway often regulate the overall pathway activity. Saal *et al.* determined PTEN (phosphatase and tensin homolog) signaling activity in breast cancers by immunmohistochemistry of the protein, and found a set of genes whose expression levels correlate with the protein level of PTEN as a PETN/PI3K pathway signature [21]. Another group independently identified a classifier for the loss of the PTEN which is composed of nine genes using xenograft models. After the identification of this classifier, they also confirmed that the protein level of IGFBP2, which changed most significantly among the nine signature genes, was inversely correlated with PTEN status and suggested that secreted plasma IGFBP2 could be a candidate biomarker for PI3K pathway activation [22].

Instead of analyzing clinical tumor samples, cancer cell lines can be used to obtain expression profiles of mutant and wild-type cells, because there are several advantages to using cell culture over tumor samples. First, the quality of microarray data of cell lines is generally higher than that of clinical tumor samples, because RNAs from tumor samples, which were often retrieved from formalin-fixed, paraffin-embedded specimens, are degraded to some extent. In addition, tumor samples used in profiling experiments may be contaminated with various types of normal cells [23] and possibly a mixture of heterogeneous tumor cells. Third, the mutation status of major oncogenes and other genes involved in oncogenic signal transduction has been determined for commonly used cell lines, and our knowledge of mutations is rapidly accumulating thanks to the Cancer Genome Project [24]. One prominent study to identify a pathway signature by leveraging expression profiles of cell lines with or without oncogenic mutations was conducted by Choi *et al.* in order to find a set of genes which represent EGFR mutation status. They analyzed the profiles of eight non-small cell lung carcinoma (NSCLC) cell lines with known EGFR mutation status to identify genes regulated by constitutive activation of the receptor [25]. Subsequently, they analyzed the expression profile of NSCLC patients by using the EGFR signature as a probe, and found that a subset of the clinical samples showed a coherent expression pattern, indicating that the EGFR mutation signature developed with cultured cell lines could potentially predict the mutation status of clinical samples.

The discovery of signature genes by mining expression profiles of cancer cells is of great significance in that the signature genes can predict the activation/inactivation status of the cancer signaling pathway more accurately compared with just looking at single gene mutations. In general, a signaling pathway is regulated by several key molecules involved in the pathway. Therefore, the deregulation of any key molecule results in similar pathway hyperactivation/inactivation. Differences in predicted pathway inactivation by the signature and real genetic status have been reported and discussed. Miller *et al*. reported that the accuracy of p53 signature classifier genes in predicting the mutation status in human breast cancer was about 85%, which is statistically significant, but that 20 out of 251 tumors were consistently misclassified by different methods [20], suggesting that other genes affect the p53 pathway. Since some tumors overexpress MDM2 or MDM4, negative regulators of p53 that exhibit a similar phenotype to p53 mutant tumors, we could speculate that the p53 signature might classify those samples into p53 defective tumors. Another example showed that the PTEN loss signature, developed from the IHC of 105 breast tumors, classified 44% of PTEN positive tumors as PTEN negative when the signature was applied to test samples [21], suggesting that PI3K pathway activity is at least partially regulated by other mechanisms, which include gain-of-function mutations in PI3KCA, and overexpression or hyperphosphorylation of PDK1, AKT1, and mTOR. Importantly, in both of these examples, signature genes show a better correlation with the prognosis of the patients than the mutation status, which means the expression pattern of signatures probably represents pathway activation more accurately than an analysis of genetic mutations.

One of the potential issues regarding the development of signature genes utilizing cell lines or clinical tumors with naturally occurring genetic mutations is the mutually exclusive relationship between mutations. For example, EGFR and KRAS mutations are reported to be mutually exclusive in non-small cell lung carcinoma [26], which means that genes selected by their correlation with EGFR mutation status may be not regulated by EGFR, but instead regulated by KRAS. In the case of EGFR and KRAS, both regulating the RAF-MEK-ERK pathway, it is difficult to discriminate genes specific to KRAS and those controlled by both KRAS and EGFR. When developing pathway signatures using heterogeneous samples, the effects of other mutations should be removed as much as possible. One of the methods to address these issues is to make use of genetically engineered mouse (GEM) models. Advances in sophisticated transgenic or knockout techniques have enabled the development of tumor models which are useful for studying the mechanisms of tumorigenesis and metastasis, and have been utilized as cancer models which mimic human clinical tumors [27]. Liver-specific knockout of the RB gene led to the identification of genes up-regulated in RB-deficient hepatocellular carcinoma (HCC) but not in RB-positive HCC [28]. The expression differences of the signature developed with the GEM model were purely derived from differences in the genetic status of RB, since they possess an isogenic genetic background. Whether or not the GEM models recapitulate human cancers is not clear, but oncogenic signaling is regulated similarly between mouse models and human cancer according to mRNA profiling analysis, as shown by Sweet-Cordeo *et al*. A comparison of a mouse lung cancer model with K-ras mutations and human lung cancer with KRAS mutations revealed that mouse signature genes were also consistently regulated in humans [29]. The GEM can provide the signature gene for the signaling pathway, which excludes the potential problem of mutually exclusive mutations, which are an intrinsic issue when using human cancer cells.

### Transient Regulation of Signaling Pathways by Exogenous Expression or Suppression

An intracellular signaling pathway can be transiently regulated by extracellular stimuli, exogenous gene activation or suppression, or the inhibition of proteins. Thus, the majority of pathway signature studies have employed transient perturbation of signal transduction by various methods. Exogenous gene expression is particularly suitable for monitoring pathways regulated by transcription factors or other proteins directly affecting the mRNA levels of target genes. Wnt signaling is canonically mediated by β-catenin and TCF transcription factors. Overexpression of dominant negative TCFs in a colorectal cell line, where the Wnt pathway is constitutively active, has identified TCF/β-catenin target genes which can be utilized as signature genes to represent the activity of the Wnt/β-catenin pathway. The significance of these genes is supported by the fact that the majority of them are upregulated in colon adenoma or carcinoma [30].

Small interfering RNAs (siRNAs), which are commonly used to silence the gene of interest, are also useful in pathway signature development. When p53-dependent expression was studied using human mammary epithelial cells and p53 positive breast cancer cell lines treated with p53 short hairpin RNA, the expression pattern of the resultant signature clearly correlated with the p53 status of breast tumors [31]. Small molecule compounds, which inhibit or activate specific target proteins, also provide opportunities to develop pathway signatures, such as EGFR inhibitor or HER2 inhibitor for growth factor signaling, and gamma-secretase inhibitor for the Notch pathway [32,  33].

Finally, for pathways stimulated by extracellular ligands, treatment of cells with the ligands is a simple but reliable method to detect expression changes. Discovering signatures with this method could reduce the number of false positive signature genes, while other pathway regulating modalities such as siRNAs or small molecule compounds may induce off-target effects and may cause artificial aberrant expression patterns which will not be observed *in vivo*. Examples of this type of study include identification of transforming growth factor-β (TGF-β) responsive genes and epidermal growth factor (EGF) signatures [34,  35].

One of the fundamental questions we have to keep in mind when developing a pathway signature is what the difference between gene mutation signatures and those identified by transient up- or downregulation of the pathway ("transient signature" hereafter) is. In some cases, transient signatures show a consistent pattern with gene mutation or amplification status [31,  35]; however, expression changes of transient signatures may not be stable in cells over the time-course of exogenous gene expression, because the changes induced by transient expression change again due to positive and negative feedback loops of the signaling pathway. Accordingly, a common problem in transient regulation is to find the appropriate time to measure expression. Currently, transient signatures have not yet been well applied to *in vivo* studies. Oncogenic pathway signatures developed from transient overexpression experiments have been correlated with sensitivity to chemotherapeutic drugs in cell lines [36,  37], but further studies will be required to confirm correlations between the signatures and the pathway status *in vivo*.

Some studies have combined more than one method to obtain reliable signatures which are not affected by differences in species or development methods. The profiling of AKT1 transgenic and wild-type mice, with or without mTOR inhibitor treatment, identified genes regulated by AKT1 expression and reversely regulated by the mTOR inhibitor rapamycin [38]. Meta-analysis which further combined the AKT1/rapamycin data with publicly available breast tumor data sets revealed that a subset of the AKT1-regulated genes, upregulated by AKT1 and decreased by rapamycin, were co-regulated in human tumors and were associated with ER negative status and poor prognosis, but the other subset downregulated by AKT1 was not [39], indicating that a combination of different methods produces a biologically more relevant gene set.

## STATISTICAL PREDICTION OF PATHWAY ACTIVITY BY SIGNTAURE GENE EXPRESSION

Practical issues in pathway signatures include how to select genes from tens of thousands of genes on a microarray and how to predict the activation status in new samples from expression data of the signature genes with appropriate statistical methods. An expression signature usually consists of up- and downregulated genes, similar to a classification, prognosis, or drug sensitivity signature. Thus, statistical methods used for gene selection and prediction are common to those used in the other types of signature analysis. For mutation status prediction, a variety of classification methods can be used, such as linear discriminant analysis, k-nearest neighbors, and support vector machine [20]. As for intracellular signaling activity, which is usually not a categorical variable like ON or OFF but rather a metric variable, scoring or p-values calculated from expression levels of all signature genes are used as a prediction parameter. As parameters, average or sum of log ratio measures and nearest centroid classifiers were found to be useful in several reports [21,  28,  40]. The KRAS expression signature, developed from a mouse tumor model, was shown to be significantly enriched in KRAS mutated human lung adenocarcinoma data sets by a rank order-based statistical test to detect subtle but significant similarity hidden in genome-scale profiles [29]. Bayesian regression with singular value composition was shown to be useful, given that signatures developed from transient expression experiments in cell lines predicted the pathway status of non-treated cell lines by this method [36,  41].

## POSITIVE AND NEGATIVE FEEDBACK LOOPS OF THE PATHWAY

The complexity of signaling network regulation is mainly derived from positive and negative feedback regulation of upstream components of the pathway by downstream effectors, and from cross-talk between pathways. In developing pathway signatures using perturbagens, negative feedback by the target or by downstream components can be problematic, because it can reverse the expression change of the signatures. For example, reversible Myc transgenic mice showed that many of the genes induced within 24 hr after Myc activation were repressed after 21 days [42], which means the signature at 24 hr is not suitable to predict constitutively active Myc signaling.

Negative feedback also exists in PI3K and MAPK pathways. It is known that p70S6K1, a phosphorylation target of mTOR, phosphorylates and inactivates IRS, leading to AKT activation. In accordance with this, mTOR inhibition induces AKT activation [43], probably due to negative feedback by p70S6K1. In the MAPK pathway, RSK inhibits ERK, which is a downstream kinase of RSK in the RAS pathway during Drosophila development [44].

The mechanism of transcriptional negative feedback in EGF signaling has been analyzed in depth by time-course experiments [45]. After twenty minutes of EGF stimulation, HeLa cells began to express immediately early genes (IEGs) such as transcription factors FOS, JUN, and EGR1, followed by the expression of "delayed early genes" (DEGs) between 20 to 240 minutes after stimulation. Transcription factors in DEGs repress the activity of AP-1 and EGR1 to attenuate their own transcription as a negative feedback. 

We have to carefully design experiments with feedback loops in mind when a pathway signature is developed. For example, although transient exogenous gene expression might clearly highlight expression changes with wider a dynamic range or larger fold-change, it might be reasonable to apply the methods only to genes such as p53 which work transiently even in cancers, the expression of which is regulated in response to DNA damage or cellular stress. For constitutively activated or inactivated genes such as Myc or PTEN, the power of transient perturbation might be limited due to the strong feedback effect of the pathway.

## APPLICATION OF PATHWAY SIGNATURES TO CANCER THERAPY

### Predicting Responses to Pathway Inhibitors by Signatures

**Fig. (2):**
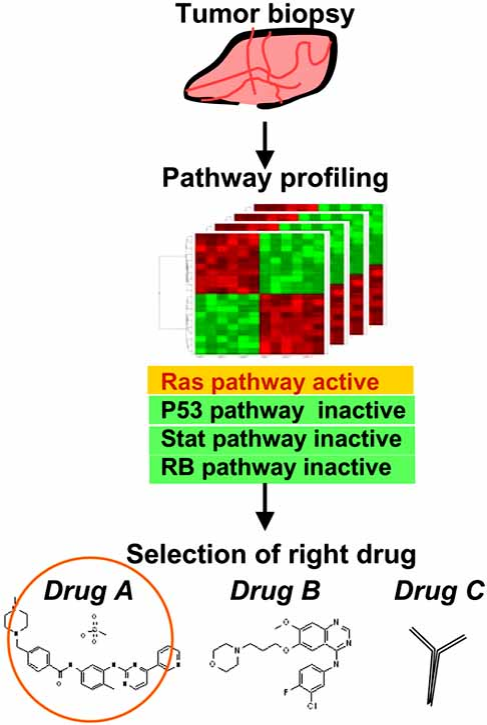
Expected application of pathway signatures to personalized medicine. In many diseases including cancer, the disorder is caused by deregulation of cellular signaling. Pathway signatures can be a novel tool to assess signaling statuses. The pathway activation/inactivation status in each patient will be monitored by the expression profile and a set of pathway signatures. Identification of the causative pathway allows us to select the "right" drug to inhibit the pathway.

The basic idea of a pathway signature is that the expression pattern of the signature for a certain oncogenic pathway can monitor the activity of the aberrant pathway even though its deregulation is caused by different types of genetic mutations in the same pathway. For instance, a pathway signature developed for PI3K might detect each instance of pathway activation caused by AKT, PTEN, or mTOR, which are located in the singular pathway. When there are two or more genes whose mutations affect the signaling, genes commonly regulated by those mutations are considered to be a pathway signature. Estimating the pathway activation level by the pathway signature will allow us to select responder patients and predict the efficacy of pathway inhibitors (Fig. **2**).

In practice, however, the effects of two different mutations in one pathway on expression profiles are usually not exactly same because of branches, convergences, or feedback loops. In anti-cancer drug development, it is of significant importance for patient stratification and the prediction of drug responses to decipher which genes are responsible for oncogenic pathway activations, and also important for developing various sets of pathway signatures fine-tuned for individual mutations in the same pathway.

**Fig. (3):**
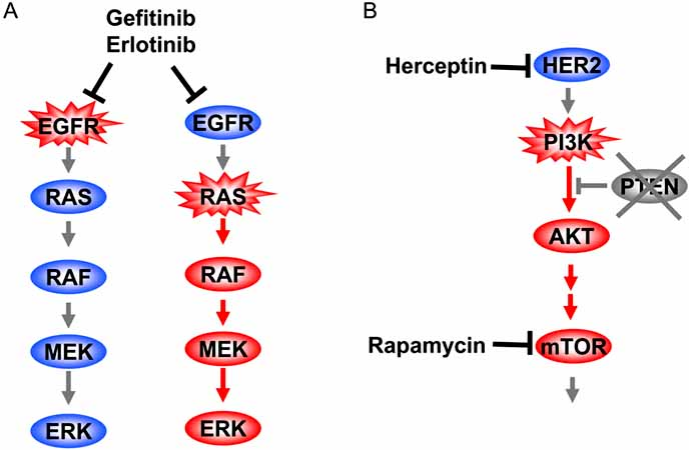
Response to pathway inhibitors of tumor cells depends on the mechanism of pathway activation. **(a)** Tumor cells with EGFR mutation are sensitive to EGFR inhibitor gefitinib or erlotinib, whereas those with RAS mutation are resistant to the same drug, because RAF-MEK-ERK signaling is not inhibited by the drugs. **(b)** When the PI3K pathway is hyperactivated by PI3K mutation or by PTEN loss, tumor cells are sensitive to rapamycin, which inhibits mTOR downstream of the pathway. However, the HER2 inhibitor Herceptin cannot stop the deregulated signal, because it does not inhibit hyperactivation downstream.

Receptor tyrosine kinases transduce extracellular signals into intracellular responses *via *PI3K and MAPK pathways, components of which are thought to be drug targets. Tumor cells whose growth depends on constitutive kinase activity of EGFR caused by mutation are sensitive to EGFR inhibitors, such as gefitinib and erlotinib, whereas those with the wild-type gene do not respond to the drugs [46,  47]. On the other hand, activating mutations in KRAS, which converts signals from EGFR into MAPK and PI3K pathways in normal cells, predict resistance to EGFR inhibitor [48,  49], because mutant KRAS can activate downstream pathways and lead to cell growth in a ligand-independent manner (Fig. **3**). Similarly, *HER2*-overexpressing breast cancer patients with PIK3CA mutation or low PTEN expression, both of which result in PI3K pathway activation, are resistant to Herceptin, a HER2 inhibitor [50]. In contrast, PTEN-deficient tumors and AKT1-induced prostate intraepithelial neoplasia are sensitive to inhibition of mTOR, which is a downstream effector of the PI3K/AKT pathway [38,  51].

These observations indicate that as a rule, inhibition of signaling downstream of the pathway activator results in increased sensitivity, whereas inhibition upstream of the activator does not. Therefore, activation of the pathway upstream of the target molecule predicts the response to the inhibitor. Based on these concepts, it is crucial to develop biomarkers which discriminate various deregulations in a single signaling pathway, or "step-specific markers", to realize personalized medicine.

There are several examples of identifying signatures which discriminate different activation mechanisms in a single signaling pathway. Activating mutations in RAS family and RAF family genes cause various cancers [52,  53]. Because KRAS and BRAF mutations are mutually exclusive in cancer, Kim *et al*. explored the difference in gene expression between the two mutant groups in colorectal cancer cell lines [54], which would be useful to classify responder/non-responders for RAS or BRAF inhibitors. Creighton *et al.* identified genes commonly regulated by overexpression of EGFR, or constitutively active HER2, RAF, or MEK in breast cell lines as a "MAPK signature", which showed hyperactivation of the MAPK pathway in estrogen receptor (ER) negative breast cancers [35]. Furthermore, they identified the expression signatures specific to EGFR or HER2 activation. Although both of the receptors stimulate downstream EGF signaling, the receptor-specific signatures revealed that the MAPK pathway is activated by either EGFR or HER2, rather than both, and that EGFR is the main driver of MAPK signaling in ER negative breast cancers. Even though the aforementioned molecules work in the same EGFR/RAS/MAPK signaling cascade, the consequent expression changes caused by the deregulation of each molecule differ from each other. Further elucidation and development of pathway signatures leads us to understand more about the similarities and differences of the functional effects of the mutations in cancer-related genes, which will enable the selection of the right drugs for the right patients.

### From Single Gene Expression Profiling to Pathway Profiling

Our knowledge about gene expression changes for various pathways is increasing as expression profile data are accumulating. With signatures for oncogenic and cancer related pathways, we will be able to classify tumors in terms of intracellular signaling and better understand the mechanisms of tumorigenesis, invasion, and metastasis, according to pathway activation patterns. Oncogenic pathway activities predicted by signatures, which have been developed by exogenous expression of Myc, Src, Ras, E2F3, and β-catenin, revealed that cancer cell lines showed different pathway activation patterns [36]. This type of pathway profiling analysis, which monitors various cancer-related pathways in parallel, is expected to be more reliable in predicting pathway activity than by using only one set of genes. With pathway profiling that deals with multiple signaling pathways simultaneously, we will be able to classify tumors based on the activation/inactivation score of each cancer-related pathway.

## INTEGRATION OF PATHWAY SIGNATURES WITH OTHER –OMICS 

### Chemogenomics

Other than pathway signatures, there are other types of genome-wide pathway analyses. Among them, expression profiling of cell lines treated with diverse drugs, often called chemical genomics or chemogenomics, can be potentially integrated with and is complementary to pathway regulation profiles. A chemical genomics study to link gene expression to drug sensitivity was first published in 2000, when more than 70,000 compounds were tested against an NCI60 cell line panel and the pre-dose mRNA profiles were determined [55]. More recently, this approach was expanded to post-dose profiles for 164 perturbagens, to identify target pathways of the drugs [56-58]. By searching a "Connectivity Map", we can identify compounds by which the pathway is up- or downregulated, using a pathway signature of interest as a query. Or conversely, new pathway signatures can be built from the data and applied to other profiles to see whether the pathway is regulated or not. Another advantage to using a Connectivity Map is that we can understand the signaling pathways for bioactive target-unknown compounds. After identifying the compound of interest with the required phenotype, we analyze the expression profile of samples treated with the compound. The expression pattern is used as a query against the vast majority of profiling data treated with compounds containing pathway specific inhibitors or the pathway signature developed for each pathway. This scheme will allow us to identify the involved pathway of target-unknown bioactive compounds.

### Proteomics

Proteomics technology also has identified a set of proteins which distinguish activated and inactivated signaling pathways. Goss *et al.* performed a phosphoproteomics analysis to identify phosphoproteins highly expressed in chronic myeloid leukemia with BCR-ABL protein [59]. The phosphoprotein signature for BCR-ABL could serve as novel disease biomarker or responsive-marker. Compared with the gene expression signature, protein markers are more proximal to the causative mutated genes. Therefore, a biological interpretation of the phosphoprotein signature is more feasible. In addition, the generality of the protein signature regardless the tissue type and cell type is expected to be high since the observed phospho-changes in the pathway are more proximal to the causative mutative genes. 

Protein microarray is also applicable to studying signaling pathways; it detects the interaction of proteins with proteins, nucleic acids, or other types of molecules [60-62]. EGFR receptors, when activated, phosphorylate each other at tyrosine residues, which in turn interact with SH2 or PTB domains of downstream proteins to transduce extracellular signals. In order to identify proteins which interact with each phosphorylated site of four EGF receptors, protein microarrays consisting of almost all of the SH2 and PTB domains in the human genome were tested against each phosphorylated site in EGFRs, and not only identified many new interactions, but unexpectedly revealed that EGFR and HER2 showed a more promiscuous pattern of interactions than HER3 as the affinity thresholds were lowered, suggesting a reason for why EGFR and HER2 are frequently overexpressed in cancers whereas HER3 is not [63]. Protein microarrays could highlight the distinctive role of receptor proteins, and monitoring the downstream protein-protein interaction or resultant protein modifications would allow us to determine the activation level of a cancer-related pathway.

### Computer Simulation

As we have discussed so far, protein or phosphoprotein signatures are proximal to the causative mutation which indicates the advantage of potential generality of the protein signature. However, the measurement of protein signatures consisting of multiplex protein markers might not be feasible for monitoring pathway activity in clinical samples. In contrast, gene signatures are somewhat distal from genetic mutations; however, multiplex measurement of signature genes will be possible (theoretically genome-wide analysis is feasible), which will generate robust prediction accuracy.

It is expected that mathematical modeling of signaling pathways will become increasingly important as genome level data increases. To understand the dynamics of EGF/nerve growth factor (NGF)-dependent ERK signaling networks, a simulation model was developed by integrating kinetics data from the literature and additional data from experiments. The simulation program predicted that a rapid increase of EGF or NGF causes transient ERK activation, whereas the final concentration of the growth factors determines the sustained activation level of the intracellular signaling pathway; subsequently the predicted patterns were validated experimentally, demonstrating the power of using simulations to leverage both mathematical theory and experimental results [64]. This study indicates the importance of quantitatively predicting the response of cells to the signal. Moreover, considering that the activation of ERK leads to phosphorylation of transcription factors and subsequent transactivation of target genes, the integration of gene expression, protein expression/phosphorylation, and mathematical modeling will be required to understand the entire mechanism from growth factor signal to cell proliferation control. Systems biology leveraging of computer simulations developed with a myriad of proteomics data and molecular profiling might allow us not only to select responder tumors/patients, but also reveal the dynamic intracellular molecular events from the initial drug-protein interaction to the subsequent cellular catastrophic events caused by the drugs.

## CONCLUSION

Systems biology is an attempt to understand the living cells as systems, rather than a collection of individual genes and proteins. The rapid accumulation of transcriptomics, proteomics, metabolomics, and other –omics data resulting from the advent of large-scale technologies has provided investigators an opportunity to study the cell and its signaling pathways as a system.

Deregulation of inter- and intracellular signaling pathways by genetic and epigenetic alterations plays a critical role in cancer initiation and progression. In the post-genome era, the complexity of the pathways is expected to be resolved by a systems biological approach instead of by studying the functions of individual genes or proteins. Pathway signatures provide us with a novel approach to understanding the mechanisms of tumorigenesis, invasion, and metastasis, and are also promising biomarkers for drug sensitivity predictions. Accumulating evidence shows that a pathway inhibitor is effective when growth of the tumor cells depends on activation of the target protein or on activation upstream of the pathway. Therefore, identification of the responsible protein is a critical issue for delivering anti-cancer drugs to appropriate patients.

In the near future, it is expected that cellular signaling pathways will be reconstructed as a mathematical models for practical use. A protein interaction study using proteomics arrays showed that quantitative measurement of the kinetics parameters for each component is important for network modeling [64]. Time-course experiments using DNA and protein microarrays will help develop more reliable network models which include negative feedback loops and cross-talk between pathways [45].

## Figures and Tables

**Table 1.** Examples of Pathway Signatures Developed by Various Methods

Pathway	Species	Materials	Method	Reference
E2F, Src, Myc, Ras, β-catenin	human	cell line	exogenous overexpression	[36]
EGFR	human	cell line	EGFR inhibitors treatment	[65]
EGFR	human	cell line	EGFR mutant vs. WT	[25]
PTEN	human	HEC-151 cells	exogeneous expression	[66]
PTEN	human	breast cancer	PTEN IHC positive vs. negative	[21]
PTEN	human	prostate cancer xenograft, glioblastoma	PTEN loss vs. positive	[22]
PTEN	mouse	intestinal polyp	conditional inactivation	[67]
AKT/mTOR	mouse	ventral prostate	AKT1 transgenic + mTOR inhibitor treatment	[38]
Myc	mouse, human	prostate cancer	Myc transgenic	[68]
γ-secretase	human	cell line	γ-secretase inhibitor treatment	[33, 69, 70]
BRAF	human	cell line	BRAF mutant vs. WT	[17]
BRAF, KRAS	human	colorectal cancer	BRAF mutant vs. KRAS mutant	[54]
KRAS	mouse	lung cancer	KRAS activation model vs. WT	[29]
RAS	human	cell line	Ras Inhibitor treatment	[82]
MAPK	human	cell line	exogeneous expression, EGF treatment	[35]
p53	human	breast cancer	p53 mutant vs. WT	[20]
p53	human	cell line	siRNA	[31]
RB	mouse	hepatocellular carcinoma	carcinogen induced RB+ vs. RB- tumor	[28]
RB	mouse	fibroblast	RB family null fibroblasts vs. WT	[71]
E2F	human	cell line	exogeneous expression	[72]
E2F	mouse	cell line	exogeneous expression	[73]
E2F	rat	cell line	exogeneous expression	[74]
TGFβ	human	cell line	DACH1 expression	[75]
TGFβ	human	cell line	TGFβ treatment	[76]
TCF	human	cell line	exogeneous expression of TCFs	[30]
β-catenin	mouse	skin	exogeneous expression	[77]
β-catenin	mouse	intestinal crypts	KO vs. WT	[78]
GLI1, GLI2	human	HeCaT keratinocyte	exogeneous expression	[79]
p53, RelA, ATM	human	cell line	siRNA	[80]
JNK	human	keratinocyte	JNK inhibitor treatment	[32]
Interferon	human	peripheral blood cells	Interferon treatment	[81]
